# Dynamic contrast-enhanced MR imaging in identifying active anal fistula after surgery

**DOI:** 10.1186/s12880-024-01257-w

**Published:** 2024-04-01

**Authors:** Weiping Lu, Xiaoyan Li, Wenwen Liang, Kai Chen, Xinyue Cao, Xiaowen Zhou, Ying Wang, Bingcang Huang

**Affiliations:** 1 Postgraduate training base at Shanghai Gongli Hospital, Ningxia medical university, No. 219 Miaopu Road, Pudong New Area, Shanghai, 200135 China; 2 Department of Radiology, Shanghai Pudong New Area Gongli Hospital, No. 219 Miaopu Road, Pudong New Area, Shanghai, 200135 China; 3 Shanghai Health Commission Key Lab of Artificial Intelligence (AI)-Based Management of Inflammation and Chronic Diseases, Sino-French Cooperative Central Lab, Shanghai Pudong New Area Gongli Hospital, No. 219 Miaopu Road, Pudong New Area, Shanghai, 200135 China

**Keywords:** Anal fistula, DCE-MRI, Activity, Granulation, Postoperative

## Abstract

**Background:**

It is challenging to identify residual or recurrent fistulas from the surgical region, while MR imaging is feasible. The aim was to use dynamic contrast-enhanced MR imaging (DCE-MRI) technology to distinguish between active anal fistula and postoperative healing (granulation) tissue.

**Methods:**

Thirty-six patients following idiopathic anal fistula underwent DCE-MRI. Subjects were divided into Group I (active fistula) and Group IV (postoperative healing tissue), with the latter divided into Group II (≤ 75 days) and Group III (> 75 days) according to the 75-day interval from surgery to postoperative MRI reexamination. MRI classification and quantitative analysis were performed. Correlation between postoperative time intervals and parameters was analyzed. The difference of parameters between the four groups was analyzed, and diagnostic efficiency was tested by receiver operating characteristic curve.

**Results:**

Wash-in rate (WI) and peak enhancement intensity (PEI) were significantly higher in Group I than in Group II (*p* = 0.003, *p* = 0.040), while wash-out rate (WO), time to peak (TTP), and normalized signal intensity (NSI) were opposite (*p* = 0.031, *p* = 0.007, *p* = 0.010). Area under curves for discriminating active fistula from healing tissue within 75 days were 0.810 in WI, 0.708 in PEI, 0.719 in WO, 0.783 in TTP, 0.779 in NSI. All MRI parameters were significantly different between Group I and Group IV, but not between Group II and Group III, and not related to time intervals.

**Conclusion:**

In early postoperative period, DCE-MRI can be used to identify active anal fistula in the surgical area.

**Trial registration:**

Chinese Clinical Trial Registry: ChiCTR2000033072.

## Background

Anal fistula is a common disease usually manifested as local pain and discharge, which is defined as the abnormal connection between the perineal skin and the anal canal [1, 2]. The cryptoglandular hypothesis is a widely recognized cause of idiopathic anal fistula, which stems from intersphincteric gland infection and its drainage obstruction [3]. Other anal fistulas are secondary to underlying causes, including Crohn’s disease, tuberculosis, pelvic infection, diverticulitis, trauma, malignancies, radiotherapy [2, 4].

Recurrence of anal fistula usually occurs due to incomplete obliteration of the fistula track and its associated elements during operation [5]. Therefore, accurate preoperative evaluation plays a critical role in the development of individualized treatment strategies and is essential for successful surgical treatment [6]. Magnetic resonance imaging (MRI) is the preferred imaging modality of diagnosing and monitoring anal fistula [7]. It provides detailed anatomical information about the anal region [8], and accurately describes the characteristics of anal fistulas, including the number and location of primary fistula tracks and secondary extensions, internal opening position, the presence of abscess, etc [2, 9, 10]. In recent years, in addition to conventional MRI sequences, some advanced sequences have been gradually applied to research on anal fistula. The volumetric contrast-enhanced three-dimensional T1-weighted (CE 3D T1) sequences with a shorter scanning time may display internal openings better than conventional two-dimensional sequences [11]. Diffusion-weighted imaging (DWI) helps to improve the diagnostic accuracy of anal fistula and perianal abscess [12, 13]. DWI includes intravoxel incoherent motion (IVIM), diffusion tensor imaging (DTI), and magnetization transfer imaging (MTI) are used to evaluate the activity of anal fistula [14–16]. Moreover, dynamic contrast-enhanced MRI (DCE-MRI) can analyze semi-quantitative and quantitative parameters to assess anal fistula activity and provide information about microvascularization [14, 17, 18].

However, compared with almost all studies focused on preoperative MRI, the application of postoperative MRI is very rare, which can evaluate surgical effectiveness like missed track or internal opening, and postoperative complications like abscess formation or recurrence track. In the early postoperative stage, MRI evaluation has been faced with challenges because of complex MRI signals, difficulties in distinguishing healing (granulation) tissue from active fistula, the presence of untreated tracks and extensions without clinical symptoms [7, 19].

Therefore, the purpose of this study was to differentiate postoperative healing tissue and active fistula by analyzing DCE-MRI semi-quantitative parameters for assessing surgical effectiveness and monitoring postoperative complications, especially in patients with complex fistula or existing clinical symptoms after surgery.

## Patients and methods

### Patients

A total of 36 consecutive patients admitted during the period of September 2018 to December 2019 were enrolled in this study, encompassing 31 men and five women aged 22 to 71 (median 39.5 ± 25.75). Thereinto, 13 patients underwent preoperative DCE-MRI but not postoperative MRI, 14 patients underwent postoperative DCE-MRI and preoperative conventional MRI (non-DCE-MRI), and 9 patients underwent both preoperative and postoperative DCE-MRI. All patients in this study were suffering from idiopathic cryptoglandular anal fistulas, while other secondary anal fistulas were excluded, such as Crohn’s disease, tuberculosis and malignancies.

### MRI examination and technique

MRI examinations were performed on a 3.0 T MR scanner (Vantage Titan, Canon Medical Systems Corporation, Japan), using a 16-channel surface coil. Patients were imaged in a head-first supine position with the center of the magnetic field on the pubic symphysis. Prior to the MRI examination, no specific bowel preparation was administrated, and no antispasmodic agent was required as a premedication. Oblique axial and coronal images were obtained by orientation perpendicular and parallel to the anal canal. For all patients, all MRI scans are performed with the same protocol (Table 1).

**Table 1:** MRI protocol

Parameters	T2WI	T1WI	T2WI	T2WI	T2WI	DCE-MRI	T1WI/Post
Sequence type	2D FSE	2D FSE	2D FSE	2D FSE	2D FSE	3D FFE	2D FSE
Acquisition plane	Sagittal	Oblique axial	Oblique axial	Oblique axial	Oblique coronal	Oblique axial	Oblique axial
Fat suppression	SPAIR	—	—	SPAIR	SPAIR	FS (Enhanced)	FS (Strong)
TR/TE (ms/ms)	4039/96	480/10	4789/80	6641/80	4039/96	3.7/1.3	630/10
Flip angle	90/120	90/160	90/160	90/160	90/130	12	90/160
Bandwidth (Hz/pixel)	195.3	244.1	244.1	244.1	195.3	488.2	244.1
Number of echo factor	15	2	17	17	15	—	2
Slice thickness (mm)	4	4	4	4	4	4	4
Intersection gap (mm)	0.4	0.4	0.4	0.4	0.4	0	0.4
Number of slices	15	26	26	26	15	26	26
Acquisition matrix	224 × 304	256 × 288	256 × 288	256 × 288	256 × 320	192 × 192	256 × 288
FOV (mm)	290 × 250	250 × 200	250 × 200	250 × 200	220 × 250	250 × 200	250 × 200
Time (min: sec)	1:25	2:04	1:21	1:53	1:49	3:46 (20 times)	1:24

*T2WI* T2-weighted image, *T1WI  *T1-weighted image, *DCE-MRI  *Dynamic contrast enhanced MRI, *FS *Fat suppression, *TR *Repetition time, *TE*  Echo time, *FOV  *Field of view, *FSE *Fast spin echo, *FFE  *Fast field echo, *SPAIR  *Spectral attenuated inversion recovery

DCE-MRI was performed using a dynamic three-dimensional T1-weighted fast field echo imaging (3D-T1-FFE) with the following sequence parameters: TR = 3.7 ms, TE = 1.3 ms, flip angle = 12 degrees, bandwidth = 488.2 Hz/pixel, slice thickness = 4 mm, intersection gap = 0 mm, number of slices = 26, acquisition matrix = 192 × 192, FOV = 250 mm × 200 mm. Consecutive imaging was composed of 20 repeated scans with a total scan time of 3 min 46 s. Starting from the second repeated scan, the contrast agent (Gadopentetate Dimeglumine Injection, Shanghai Xudong Haipu Pharmaceutical Co., Ltd, China) was administered intravenously at a dose of 0.1 mmol/kg (0.2 ml/kg) of body weight through an 22-gauge intravenous catheter with an automated injection pump (Optistar Elite, Liebel-Flarsheim Company LLC, USA). After bolus injection (3 mL/sec) of the contrast agent, a 15 mL saline solution was immediately injected at the same rate. An Oblique axial T1-weighted fast spin echo (FSE) sequence with fat suppression was performed in the end.

### Image analysis

#### St James’s University Hospital classification

The St James’s University Hospital classification is a MR imaging-based grading system that can be easily applied and can accurately assess the relationship between primary fistula tracks, secondary extensions, abscesses and normal anatomical structures. The system is divided into five groups [20]: Grade 1, simple linear intersphincteric fistula; Grade 2, intersphincteric fistula with abscess or secondary track; Grade 3, transsphincteric fistula; Grade 4, transsphincteric fistula with abscess or secondary track within the ischiorectal fossa; Grade 5, supralevator and translevator disease.

#### Normalized signal intensity

Based on the quadriceps muscle as a reference organ, the normalized signal intensity (NSI) was defined as a ratio of fistula wall to quadriceps muscle signal on the oblique axis fat-suppressed T2-weighted image.

#### Semi-quantitative parameters of DCE-MRI

**Fig. 1:**
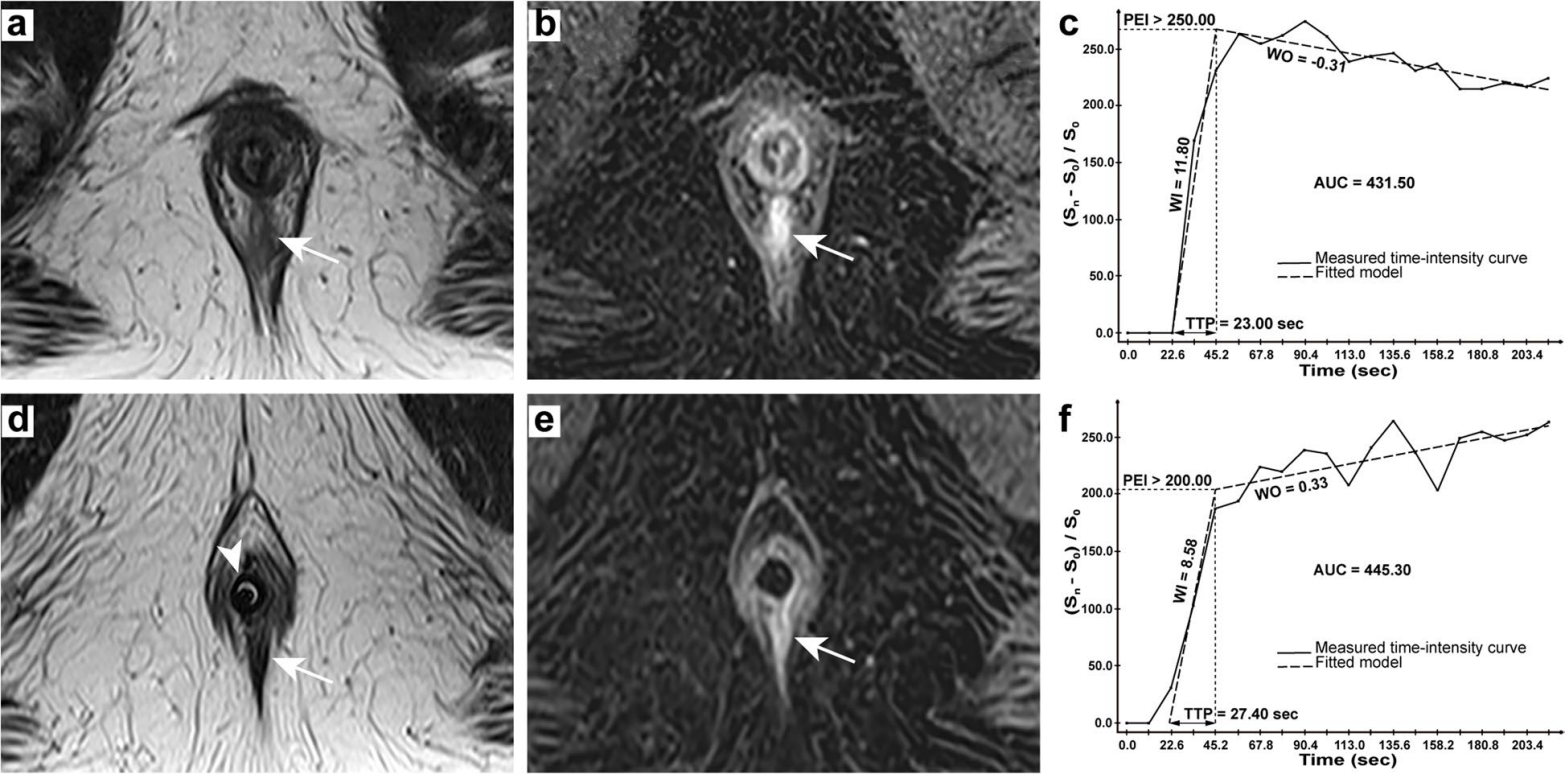
42-year-old man presenting a simple linear intersphincteric fistula. Preoperative MRI: axial T2-weighted image (**a**) showing the active fistula in hyperintensity (arrow), axial DCE image (**b**) showing early strong enhancement of the fistula (arrow) and TIC with parameters (**c**); Postoperative MRI: axial T2-weighted image (**d**) showing the healing tissue in hypointensity (arrow) and a balloon catheter (arrowhead), axial DCE image (**e**) showing progressive enhancement of the lesion (arrow) and TIC with parameters (**f**)

All DCE-MRI semi-quantitative parameters are based on the shape and structure of the time-intensity curve (TIC), including wash-in rate (WI), wash-out rate (WO), time to peak (TTP), peak enhancement intensity (PEI), area under the curve (AUC), were calculated on the imaging workstation (Myrian V1.12, Intrasense, France) (Fig. 1). The concentration computation method used to generate parametric maps was set to $({\text{S}}_n-{\text{S}}_0)/{\text{S}}_0$ (a relative normalization using the baseline) the MR series. Regions of interest (ROIs) were placed on the fistula wall and healing tissue. Three areas with the highest enhancement (usually 17–19 times in 20 repeated scans) were measured, and the average value was taken as the final value, with a median of 3.84 mm^2^.

### Standard of Reference

Clinical symptoms, MRI manifestations, results of anoscopic examination, intraoperative findings as well as pathological results were used as diagnostic criteria for active fistula, and the interval between preoperative MRI examination and operation was less than three days. If there were no clinical symptoms during the postoperative MRI reexamination and no recurrence in a clinical follow-up for at least six months after the reexamination, it was classified as postoperative healing (granulation) tissue. If there were clinical symptoms and suspected residue or recurrence of fistula track on MRI images, which was later confirmed by surgery and pathology, it was classified as active fistula.

### Statistical analysis

Descriptive statistics were presented as median ± interquartile range for non-normally distributed continuous data and as numbers and percentages for categorical variables. The difference in classifications of anal fistula between two predefined groups was analyzed using Mann-Whitney test. Spearman’s correlation test was performed between time intervals and parameters of postoperative MRI reexaminations. The differences in MRI parameters between four predefined groups were quantitatively compared using Kruskal-Wallis test, and then receiver operating characteristic (ROC) curves were used to evaluate the diagnostic ability, including the optimal cutoff, area under curve (AUC) with 95% confidence interval (CI), sensitivity, and specificity. ROC curves were conducted with MedCalc 19.2 (MedCalc Software Ltd, Ostend, Belgium; https://www.medcalc.org), and other statistical analyses were performed using SPSS 26.0 (IBM Corporation, Chicago, USA). A two-tailed *p* value of less than 0.05 is considered to indicate statistical significance.

## Results

### Characteristics of groups

According to the standard of reference, we divided all subjects into the active fistula group (Group I) and the postoperative healing (granulation) tissue group (Group IV), and then divided the latter into two groups according to the time interval of 75 days after operation, namely Group II (less than or equal to 75 days) and Group III (more than 75 days). All preoperative fistulas and one postoperative residual fistula, which originated from 14 patients who underwent postoperative DCE-MRI and were reconfirmed surgically and pathologically, were classified as active fistulas, and the rest of the postoperative ones were classified as postoperative healing (granulation) tissue. Accordingly, Group I had a study population of 23 patients aged 31 to 71 years (median 43.5 ± 26.5), and Group IV had 22 patients aged 22 to 71 years (median 42 ± 27.5). The time interval between operation and postoperative MRI reexamination ranged from 50 to 363 days (median 84.5 ± 104.5) in Group IV, 50 to 73 days (median 59 ± 10) in Group II and 96 to 363 days (median 161 ± 154) in Group III.

### Anal fistula classification

**Fig. 2:**
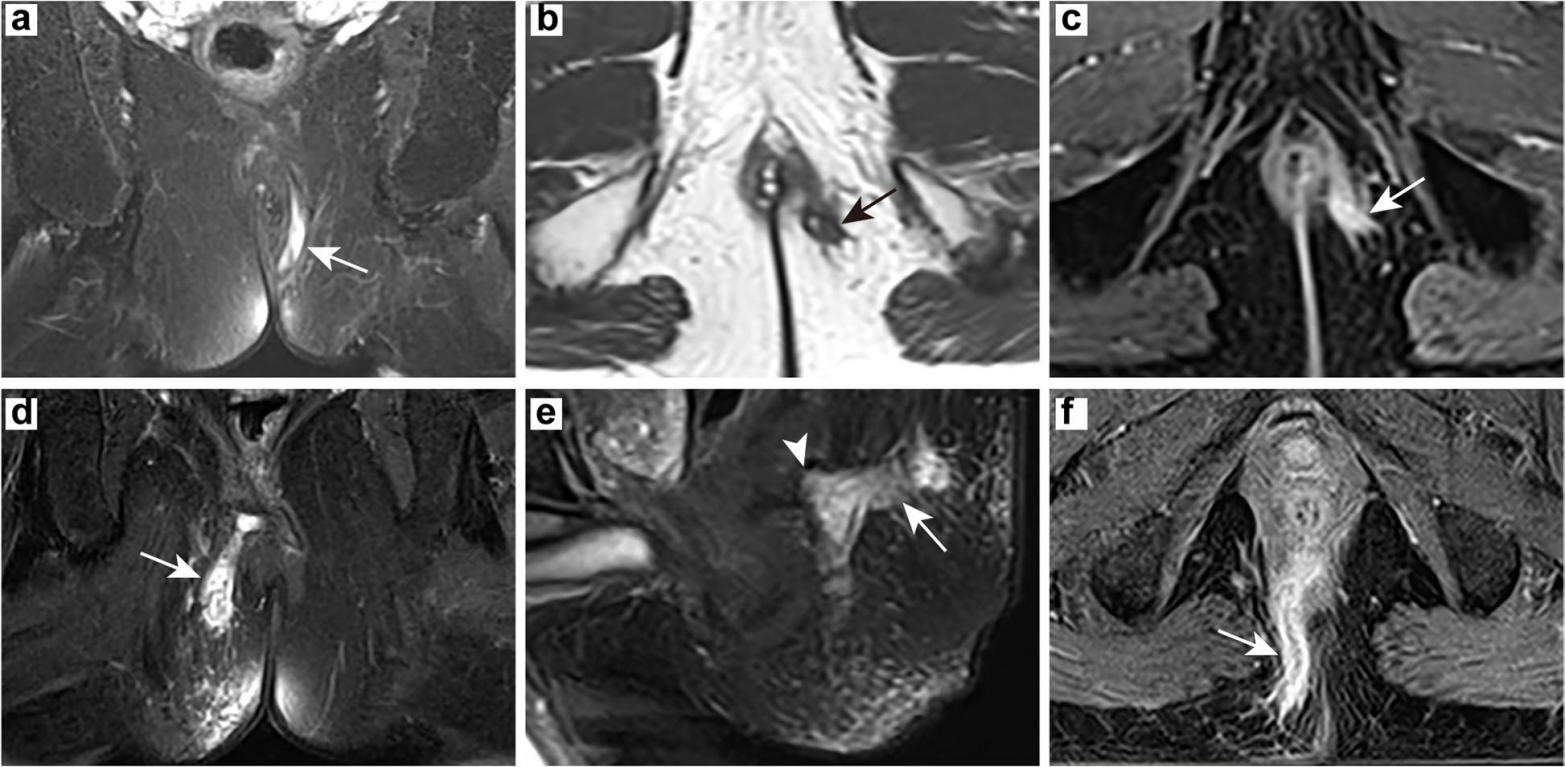
St James’s University Hospital Classification. **a**-**c** 65-year-old male from Group IV, Grade 3 of classification: oblique coronal T2WI fat suppression sequence (**a**), oblique axial T2WI sequence (**b**) and oblique axial T1WI enhancement sequence (**c**) showing the transsphincteric fistula (arrow); (**d**-**f**) 54-year-old male from Group IV, Grade 4 of classification: oblique coronal T2WI fat suppression sequence (d) and oblique axial T1WI enhancement sequence (**f**) showing the transsphincteric fistula (arrow), sagittal T2WI fat suppression sequence (**e**) showing the main fistula (arrow) and secondary track (arrowhead)

The St James’s University Hospital classification of the active fistula group (Group I) was based on MR images performed at the time, while that of the postoperative healing (granulation) tissue group (Group IV) referred to its preoperative MRI classification. Classifications of the two groups were mainly Grade 1 and Grade 2 (47.83%, 47.83% and 40.91%, 40.91%, respectively), and there was no statistical difference between the two groups (*p* = 0.368) (Table 2; Fig. 2).

**Table 2:** St James’s University Hospital Classification of anal fistulas after MRI

	Total	Classification				*p*
	n	Grade 1	Grade 2	Grade 3	Grade 4	
Active fistula	23	11(47.83%)	11(47.83%)	1(4.34%)	0(0.00%)	0.368
Postoperative healing/granulation tissue	22	9(40.91%)	9(40.91%)	2(9.09%)	2(9.09%)	

### Quantitative analysis of parameters

**Fig. 3:**
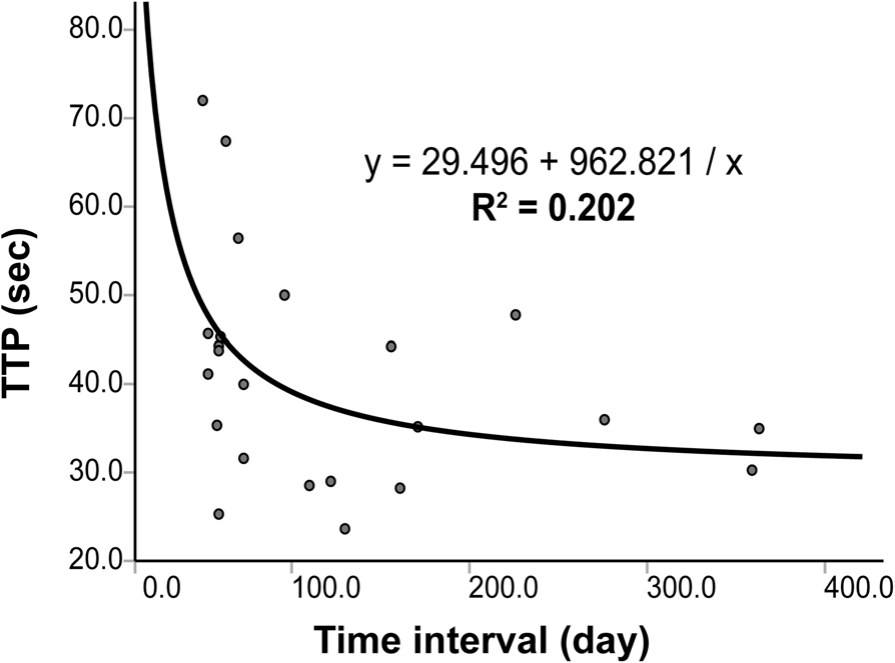
Curve fitting. Diagram shows correlation between time interval and TTP with an inverse fitting method (R^2^ = 0.202)

By correlation analysis and curve fitting, we found that all MRI parameters had no clear correlation with time intervals (Table 3), with a correlation coefficient R2 of 0.202 for TTP (Fig. 3).

**Table 3:** Rank correlation analysis between time intervals and parameters of postoperative MRI reexamination

		WI	WO	TTP	PEI	AUC	NSI
Time interval	r_s_	0.000	0.001	-0.399	-0.259	-0.260	-0.009
	*p*	0.998	0.997	0.059	0.232	0.231	0.996

**Fig. 4:**
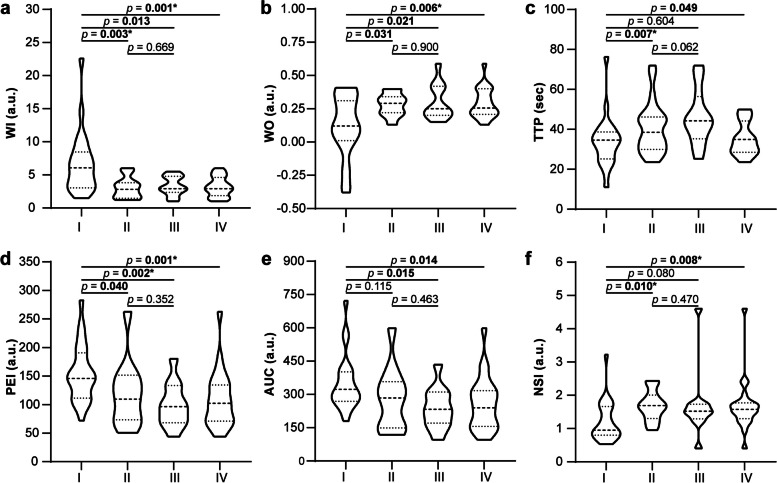
Outcomes of quantitative analyses. Differences were analyzed between Group I and II, III, IV, and between Group II and III in wash-in rate (**a**), wash-out rate (**b**), time to peak (**c**), peak enhancement intensity (**d**), area under the curve (**e**), and normalized signal intensity (**f**). Bold values are statistically significant (*p* < 0.05); * indicate values of *p* < 0.01

Detailed MRI parameter values of the four groups are presented in Table 4. To begin with, WI (6.07 ± 5.42 vs. 2.82 ± 2.36, *p* = 0.003) and PEI (145.57 ± 79.30 vs. 109.57 ± 78.10, *p* = 0.040) were significantly higher in Group I than in Group II, while WO (0.12 ± 0.30 vs. 0.29 ± 0.12, *p* = 0.031), TTP (34.60 ± 13.50 s vs. 44.30 ± 21.10 s, *p* = 0.007), and NSI (0.95 ± 0.86 vs. 1.69 ± 0.71, *p* = 0.010) were significantly lower. In addition, WI (6.07 ± 5.42 vs. 2.92 ± 2.44, *p* = 0.013), PEI (145.57 ± 79.30 vs. 96.37 ± 65.20, *p* = 0.002) and AUC (322.73 ± 132.47 vs. 232.93 ± 140.10, *p* = 0.015) were significantly higher in Group I than in Group III, while WO (0.12 ± 0.30 vs. 0.25 ± 0.22, *p* = 0.021) was significantly lower. Finally, all MRI parameters showed statistical differences between Group I and Group IV, but not between Group II and Group III. The above results are shown in Fig. 4.

**Table 4:** Summary of quantitative analyses

Parameters	Active fistula	Postoperative healing/granulation tissue		
	I (*n* = 23)	II ≤ 75 days (*n* = 11)	III > 75 days (*n* = 11)	IV Total (*n* = 22)
WI (a.u.)	6.07 ± 5.42	2.82 ± 2.36	2.92 ± 2.44	2.92 ± 2.76
WO (a.u.)	0.12 ± 0.30	0.29 ± 0.12	0.25 ± 0.22	0.26 ± 0.19
TTP (sec)	34.60 ± 13.50	44.30 ± 21.10	34.97 ± 15.70	38.55 ± 16.24
PEI (a.u.)	145.57 ± 79.30	109.57 ± 78.10	96.37 ± 65.20	102.15 ± 63.10
AUC (a.u.)	322.73 ± 132.47	283.50 ± 209.03	232.93 ± 140.10	239.93 ± 161.26
NSI (a.u.)	0.95 ± 0.86	1.69 ± 0.71	1.52 ± 0.44	1.57 ± 0.47

*WI *Wash-in, *WO *Wash-out, *TTP *Time to peak, *PEI *Peak enhancement intensity, *AUC *Area under the curve, *NSI *Normalized signal intensity

I = the group of active fistula, II = the group of postoperative healing tissue within 75 days, III = the group of postoperative healing tissue over 75 days, IV = the group of postoperative healing tissue for total days

**Fig. 5:**
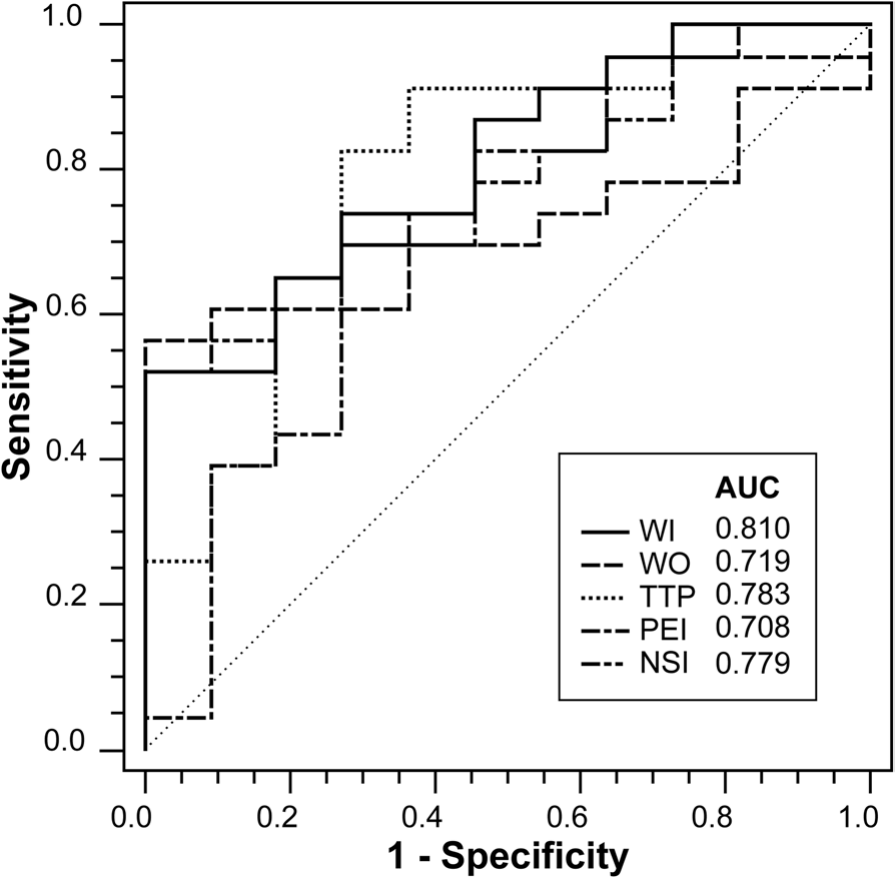
ROC curves. Graph shows ROC curves for WI, WO, TTP, PEI, NSI in the differentiation between active fistula and postoperative healing (granulation) tissue

As shown in Table 5; Fig. 5, ROC curve analysis is used to assess the ability of parameters to distinguish active fistula from postoperative healing (granulation) tissue in Group I and II. WI had a maximum AUC of 0.810 (95% CI: 0.639, 0.924) with the optimal cutoff of 6.033, and provided 52.17% sensitivity, 100.00% specificity. The second was TTP, which had an AUC of 0.783 (95% CI: 0.608, 0.905) with the optimal cutoff of 39.967 s, and provided 82.61% sensitivity, 72.73% specificity. PEI had a minimum AUC of 0.708 (95% CI: 0.527, 0.850) with the optimal cutoff of 131.567, and provided 69.57% sensitivity, 72.73% specificity.

**Table 5:** ROC analysis of parameters for the differential diagnosis between active fistula and postoperative healing (granulation) tissue (≤ 75 days)

Parameters	Optimal cutoff	AUC(95%CI)	*p*	Sensitivity(%)	Specificity(%)
WI (a.u.)	> 6.033	0.810(0.639, 0.924)	< 0.0001^*^	52.17%	100.00%
WO (a.u.)	≤ 0.120	0.719(0.539, 0.859)	**0.0118**	56.52%	100.00%
TTP (sec)	≤ 39.967	0.783(0.608, 0.905)	0.0017^*^	82.61%	72.73%
PEI (a.u.)	> 131.567	0.708(0.527, 0.850)	**0.0456**	69.57%	72.73%
NSI (a.u.)	≤ 0.949	0.779(0.604, 0.902)	0.0004^*^	52.17%	100.00%

Bold values are statistically significant (*p* < 0.05); ^*^ indicate values of *p* < 0.01

## Discussion

To our knowledge, this study is the first to evaluate healing (granulation) tissue by postoperative DCE-MRI, to identify active fistula. We found the parameters between them were significantly different, especially WI, WO, TTP, PEI and NSI were used to distinguish between healing tissue and active fistula within 75 days after surgery, which had certain diagnostic efficacy, with AUC of 0.810, 0.719, 0.783, 0.708, and 0.779, respectively. This has provided quantitative and visible information for surgeons to assess surgical outcomes and perform postoperative follow-ups.

As per the classification by St James’s University Hospital, which could be easily accepted by radiologists and could show detailed information to surgeons, most of the anal fistulas in Group I and Group IV were classified as simple intersphincteric fistulization (Grade I: 47.83%, 40.91%; Grade2: 47.83%, 40.91%). Garg [21] analyzed the correlation between the implementation of fistulotomy and grades in different classifications, and proposed a new classification. Nevertheless, all of the patients recruited in this study underwent fistulectomy and had a good prognosis.

DCE-MRI has been used primarily for the evaluation of anal fistula activity. Horsthuis et al. [17] found that absolute pixel counts of TIC shape types showed weak to moderate correlations with perianal disease activity index (PDAI) in perianal Crohn’s disease. Ziech et al. [18] reported that activity of perianal Crohn’s disease correlated with semi-quantitative parameters (maximum enhancement and initial slope of increase) but not with quantitative parameters (K^trans^ and υ_e_). Lefrancois et al. [14] showed that brevity of enhancement (a semi-quantitative parameter defined as the time difference between wash-in and wash-out) was significantly different between active and inactive fistulas (*p* = 0.02), which combined with IVIM-DWI improved the diagnostic accuracy of fistula activity. In contrast, we analyzed semi-quantitative parameters of DCE-MRI and NSI of non-enhanced MRI in preoperative and postoperative examinations.

Preoperative MRI facilitates the management of anal fistula surgery and the reduction of recurrence rate [22, 23], while postoperative MRI can accurately and intuitively evaluate surgical outcomes and complications, especially in people with complex fistula and apparent clinical healing (asymptomatic) [19]. A previous study demonstrated that the difference between healing (granulation) tissue and active fistula was difficult within 8 weeks, and an MRI scan was recommended after 12 weeks [19]. The European Society of Gastrointestinal Abdominal and Radiology (ESGAR) recommended an MRI examination four weeks after surgical intervention because of the difficulty in distinguishing postoperative cavities from untreated extensions [7]. However, our study showed differences in semi-quantitative parameters of DCE-MRI and NSI between active fistula and postoperative healing tissue. In particular, there were significant differences in parameters except AUC in early postoperative period (≤ 75 days, median 59 ± 10). Furthermore, we found no correlation between postoperative time intervals and parameters, and no significant difference between parameters of the two groups (Group II and III) bounded by 75 days, and then we speculated that uneven distribution of time intervals was one of the possible factors contributing to the two results.

This study has several limitations. First, the study was based on a relatively limited sample size, with a particular lack of postoperative residual and recurrent fistula tracks. In addition, we only included idiopathic cryptoglandular anal fistulas, hoping for further research on other secondary fistulas, such as Crohn’s disease. Second, ROI in this study did not cover the entire fistula wall and healing tissue, but was achieved by averaging the three most significant regions. ROI placement methods and DCE-MRI scanning protocols vary from study to study, both of which are common problems in current research without expert consensus. Third, we have analyzed only semi-quantitative parameters, rather than quantitative parameters related to tissue’s pathophysiological properties. However, results of the latter are influenced by selection of models, determination and measurement of arterial input function (AIF) [24].

## Conclusion

In this study, we evaluate healing (granulation) tissue by postoperative DCE-MRI to identify active fistula. The results indicates that DCE-MRI can be used to differentiate active anal fistula from healing or granulation tissue, especially in the early stage after surgery (approximately 60 days), which will provide a visual and quantitative method for surgeons to evaluate surgical outcomes and monitor complications. The data suggests that postoperative DCE-MRI parameters have the potential to serve as an imaging biomarker for predicting anal fistula surgery prognosis, which warrants further comprehensive investigation. Moreover, when combined with prevalent artificial intelligence techniques, these parameters are anticipated to present an efficient approach to the management of anal fistula treatment.

## Data Availability

The datasets used or analysed during the current study are available from the corresponding author on reasonable request.
